# Acceptability of government measures against COVID-19 pandemic in Senegal: A mixed methods study

**DOI:** 10.1371/journal.pgph.0000041

**Published:** 2022-04-25

**Authors:** Valéry Ridde, Babacar Kane, Ibrahima Gaye, Mouhamadou Faly Ba, Amadou Diallo, Emmanuel Bonnet, Zoumana Traoré, Adama Faye

**Affiliations:** 1 CEPED, IRD-Université de Paris, ERL INSERM SAGESUD, Paris, France; 2 Institut de Santé et Développement (ISED), Université Cheikh Anta Diop, Dakar, Senegal; 3 IRD (French Institute for Research on Sustainable Development) Résiliences / PRODIG, French National Research Institute for Sustainable Development, Bondy, France; 4 CloudlyYours, Morangis, France; Universitas Gadjah Mada, INDONESIA

## Abstract

While the first case of COVID-19 was declared on March 2 2020 in Senegal, the government banned the attendance of places of worship on 14 March, as a first measure. On March 23, it introduced a curfew, a ban on movement between regions, and the closure of markets. The objective of this study is to measure and understand the acceptability of these four governmental measures as well as the level of public trust in the state to fight the pandemic. We carried out a mixed-method research. The acceptability variables were defined using the theoretical framework of acceptability (TFA). At the quantitative level, we carried out a telephone survey (June/July 2020) at the national level (n = 813) with a sampling strategy by marginal quotas. We conducted a qualitative survey (August/September 2020) with a nested sample (n = 30). The results show a relatively high acceptability of the measures but a heterogeneity of responses. People considered curfews to be much more important (85.7% [83.2%; 88.0%]) than the closure of places of worship (55.4%; [51.9%; 58.7%]), which is least in line with the values and positive affective attitude. Several positive unintended effects of the curfew were stated (security and social/family cohesion). People over the age of 60 have more confidence in the government to fight the pandemic than people under the age of 25, although not significant (7.72 ± 3.12 vs. 7.07 ± 3.11, *p* = 0.1); and they are more in favour of the closure of places of worship. The more regions are affected by the pandemic, the less confidence respondents report in the government and the less they perceive the measures as effective. The results confirm the importance of government communication and trust in the state to strengthen the acceptability of pandemic measures. Important differences in acceptability show the need to adapt measures and their explanations, instead of unqualified universal action.

## Introduction

In Africa, many voices were quickly raised in early 2020 to ask whether governments and health systems were ready to deal with the COVID-19 pandemic that was sweeping across Europe after starting in China [1, 2]. It was predicted that the African continent would account for 22% of the world’s population infected with CoV-2-SARS in the first year of the pandemic, with about 150,000 deaths, including just over 2,200 in Senegal [3]. Yet Africa was well prepared according to the African Centres for Disease Control and Prevention [4]. Indeed, most African countries reacted very quickly to the announcement of the epidemic in China [5] and long before it was declared a pandemic by the WHO on March 11 2020. In French-speaking West Africa, for example, several countries had prepared themselves even before the first case occurred, and many closed their borders or organised screening very quickly [5].

In all countries of the world, multiple forms of government measures have been implemented. Many studies have previously modelled their potential effects and then the reality of their effectiveness [3, 6, 7]. There has been relatively little research to measure and understand the social acceptability of these measures, particularly in Africa. In Europe and China, studies show the important role of knowledge and vulnerability to the disease, as well as confidence in the government in the acceptance of measures to combat the COVID-19 pandemic [8–12]. In addition, political science research has long shown the importance of consistency in government measures and choices to promote their acceptability [13].

Understanding how people perceive the response to the COVID-19 pandemic is essential for planning and adapting public health interventions formulated by governments. This understanding is a research priority identified by the Global Research Forum for COVID-19 [14], and essential to support government decision-making in Africa [15].

Furthermore, few studies of the social acceptability of measures organised by an African country using a sound conceptual approach and a national sample are available. Studies in the Democratic Republic of Congo, Egypt, or Nigeria [16–18] centrally addressed acceptability and did not use a sound conceptual approach. Conceptual reflections on acceptability remain as rare as their applications [19, 20]. However, the use of conceptual frameworks is essential for research into public health interventions [21]. The methodological challenges of such a survey in Africa are significant [22, 23] as there is no national phone sampling frame. Thus many web-based surveys of knowledge about the disease [17] or government responses [24] continent have significant methodological limitations.

The objective of this article is to present the results of research using mixed methods to measure, and then understand, the acceptability of the government measures put in place in Senegal at the beginning of the COVID-19 pandemic as well as the confidence of the population in the state to fight against this pandemic.

## Materials and methods

### Context of the study and government measures studied

The research took place in Senegal which is composed of 14 regions and 45 departments for an estimated total number of 16,705,608 inhabitants (of which 50.23% are women) according to the National Agency for Statistics and Demography (ANSD) in 2020. The COVID-19 pandemic hit the country in March 2020 with, between March 2 and October 1st 2020, an estimated attack rate of 94.75 per 100,000 people, a case fatality rate of 2.07, and 1177 PCR tests per 100,000 people carried out [5].

Although Senegal had its first confirmed COVID-19 case on March 2 2020, it did not wait until that date to act. An extraordinary meeting of the national epidemic management committee was held on January 20 2020 [25]. The first significant government measure was a ban on gatherings of people and attendance at places of worship on March 14. On March 23, three important measures were decided: curfews (whose times will vary over time); a ban on travel between regions; and the closure of markets. The reopening of places of worship took place on May 11 for voluntary institutions, the restriction on movement between regions was lifted on June 4, and the curfew and closures of markets (except for one day a week at the beginning) were lifted on June 29.

### Research design

This study is an explanatory sequential mixed methods specification where the qualitative data allow the results of the analysis of previously collected quantitative data to be understood [26]. The writing of the article followed the quality criteria proposed by the Mixed Methods Appraisal Tool [27].

**Fig 1:**
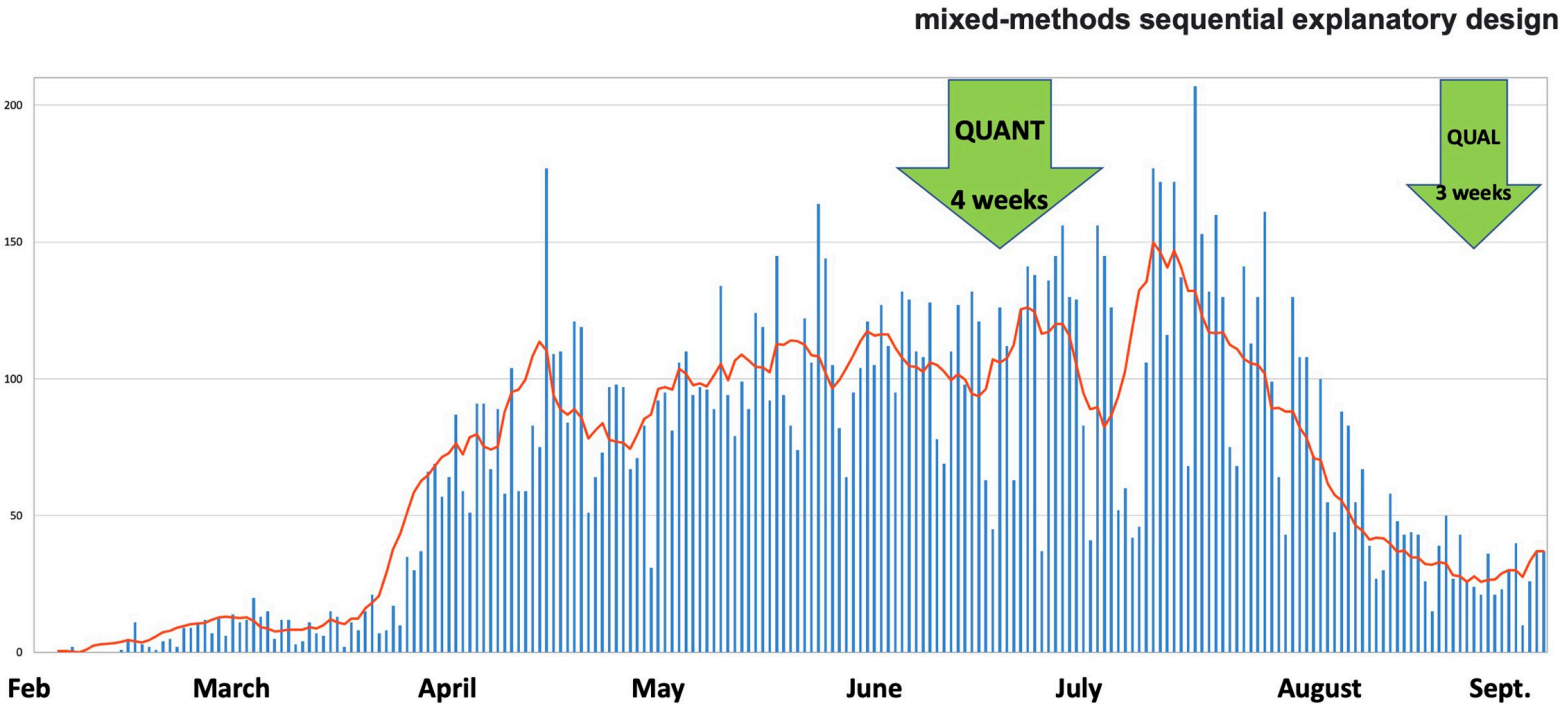
Evolution of the number of cases in Senegal and date of completion of the two surveys.

The epidemiological evolution as well as the dates on which quantitative (from June 11 to July 10) and qualitative (from August 24 to September 16) data collection was carried out are shown in Fig 1.

### Populations

The quantitative cross-sectional and descriptive study used a sampling strategy based on marginal quotas [28]. This method is relevant in emergency situations such as the COVID-19 pandemic with sample sizes below 3,000 [28, 29]. The quota sampling method can be as accurate as random sampling [30] if not better if the sample size is small [29, 31]. The following variables were used to define quotas: age, gender, and region. The last general population census (2013) was used as a reference.

We used the *Random Digit Dialing* (RDD) method to select telephone numbers. A computer program randomly generated a list of unique telephone numbers (n = 30,603) following the national numbering plan and respecting the market shares of the telephone operators. A second computer programme sent an SMS to the previous list to provide information about the project (including ethical issues) and warn subscribers that they would be likely to be called. This programme identified valid numbers by the delivery status of the SMS. This resulted in a new list of numbers assumed to be valid (n = 10,931; 35.72%). This list was then injected into a *Reactive Auto Dialer* (RAD) to trigger calls in an automatic and optimised way. When the outgoing call was picked up (n = 6,576; 60.16%), the platform automatically detected whether it was a human, an answering machine, or if there was any doubt. At this point, an audio greeting in the national language (Wolof) was played to the caller and the person was put in touch with an interviewer (n = 1441; 21.91%) based in Dakar who explained the research and asked for consent to participate (S6 Fig). Five interviewers speaking six languages (French, Diola, Wolof, Sérére, Pulaar, Soninké) carried out the data collection after three days of training.

The qualitative sample consists of 30 people, nested within the final quantitative sample (n = 813). The selection of these 30 persons followed the same stratification as the quantitative sample in order to have a diversity of points of view. These individuals were drawn at random from the quantitative sample according to this stratification and replacing individuals when they refused to participate or could not be reached. We conducted 28 of the 30 interviews in Wolof.

### Data collection instruments

The variables in the quantitative questionnaire concerning the acceptability of government measures were defined on the basis of the theoretical framework of acceptability (TFA) [19]. On the basis of an analysis of 43 reviews, the TFA proposes to measure acceptability with regard to seven dimensions: affective attitude, burden, perceived effectiveness, ethicality, intervention coherence, opportunity costs, and self-efficacy. Each of the constructs was to be judged by the individuals on a Likert scale concerning their degree of agreement from 1 (completely) to 5 (not at all). Apart from curfew, the other three measures had been removed at the time of our survey, so we measured retrospective acceptability [19]. The curfew was cancelled in the middle of our survey. Thus, the question was reworded from June 30 to move from contemporary acceptability to retrospective acceptability. S1 Table shows that there is no significant difference for five of the seven dimensions of acceptability in the responses before and after the rewording of the question. Using an internal consistency parameter (Cronbach alpha >0.7) and item response models, the analysis of the seven component constructs shows that the items and components are related; acceptability is measured as a unidimensional underlying concept.

The level of concern about the pandemic and confidence in the government to address it was measured on a scale of 0 to 10 by respondents. The authors collected the attack rate of the epidemic with government data (www.covid19afrique.com). The authors also collected individual characteristics of the respondents: age, gender, department, socio-economic level and education level.

The qualitative interviews were guided by the results of the quantitative analyses based on the following approach (S1 Text). First, the research team met in early August 2020 to conduct preliminary descriptive analyses of the quantitative data. We discussed the main differences in perceptions between individuals and between measures. The aim of this purely descriptive analysis was to avoid waiting for the complex statistical analyses (to be published later) to organise the qualitative research phase that would allow people to express themselves on relatively recent events. Then, the qualitative collection took place at the end of August and beginning of September 2020, drawing on the TFA, in order to better understand the most important variations observed in the quantitative results between measures and/or between individuals for certain measures (S1 Text).

### Analyses

For all quantitative variables, we conducted descriptive statistics (mean, median, standard deviations, confidence interval), and used Wilcoxon Mann-Whitney (confidence and gender) and Chi-square (acceptability across the two age groups) tests to compare the differences between certain groups of respondent variables and between the seven dimensions of acceptability of the four government measures. The qualitative variables were described with proportions and their 95% confidence intervals. For the correlation between attack rate and confidence across regions, we used the Spearman correlation.

For the qualitative data, the interviews were transcribed in full in French. A manual content analysis [32] was carried out for each of the questions asked with regard to the differences revealed by the quantitative analyses. Where relevant, differences in perceptions according to individuals were highlighted in the presentation of the results.

The integration of data, a principle at the heart of mixed methods [26], was carried out from a meta-inference perspective [33] when interpreting the results in the discussion. According to the mixed-method approach, divergences and convergences are put into practice in the presentation of the results [34].

### Ethics approval and consent to participate

Both surveys were carried out by telephone in view of the challenges related to the COVID-19 pandemic to respect the barrier gestures, not to put people at risk but also to have a national quantitative sample at lower costs. All individuals were informed of the ethical issues and were given the opportunity to withdraw from the study at any time. They all agreed to participate through a verbal informed consent recorded by the interviewer under the supervision of the researchers. No minors participated in this research. The research was accepted by Senegal’s national health research ethics committee (SEN/20/23) which approved the oral consent method.

## Results

The characteristics of the quantitative sample are presented in Table 1. From the 1441 completed forms (including refusals and out of quota persons), 813 persons make up the final sample. The ratio of completed forms to calls answered is 13.75% (904/6576).

**Table 1:** Characteristics of the quantitative sample (n = 813).

Region	Under 25 years old		25–59 years old		60 years old and over		Total
	M	F	M	F	M	F	
**Dakar**	28	28	83	84	12	12	246
**Diourbel**	10	13	25	33	5	6	92
**Fatick**	8	6	12	9	2	3	40
**Kaffrine**	4	2	10	5	2	2	25
**Kaolack**	12	7	17	19	3	0	58
**Kédougou**	0	0	3	1	0	0	5
**Kolda**	4	3	12	6	2	0	27
**Louga**	10	10	16	11	3	0	50
**Matam**	7	2	9	6	2	0	26
**Saint-Louis**	12	7	18	11	3	0	51
**Sédhiou**	2	1	7	3	0	0	13
**Tambacounda**	7	2	13	9	2	0	33
**Thiès**	20	16	36	39	7	2	120
**Ziguinchor**	6	1	9	9	1	1	27
**TOTAL**	130	98	270	244	44	26	813

The characteristics of the people interviewed for the qualitative part are presented in Table 2. The interviews lasted between 18 and 56 minutes with an average of 37 minutes.

**Table 2:** Characteristics of the qualitative sample (n = 30).

	Dakar		Outside Dakar		Total
	Man	Woman	Man	Woman	
Under 25 years old	1	1	3	2	7
Between 25 and 59 years old	3	3	7	6	19
Over 60 years old	1	1	1	1	4
**Total**	**5**	**5**	**11**	**9**	**30**

### The relative importance of measures

**Fig 2:**
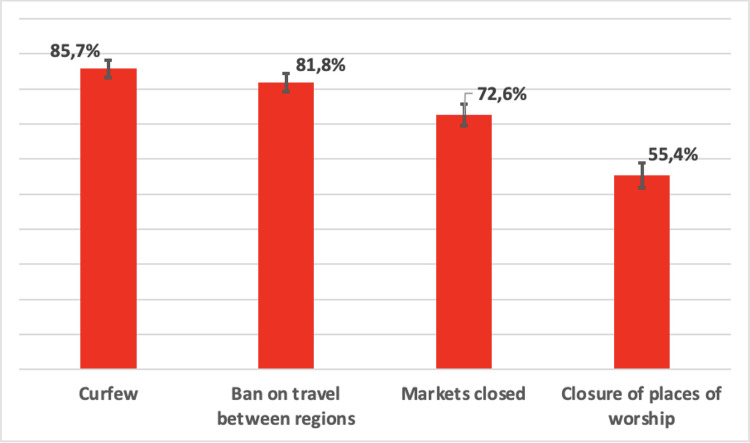
Importance of the four measures.

In the context of the pandemic, Fig 2 shows that people considered curfews to be much more important than the closure of places of worship. Moreover, the latter measure was the one that was least in line with respondents’ personal values and their affective attitude were the least positive towards it (S1 and S2 Figs).

Qualitative data confirm the low acceptability of the closure of places of worship, but opinions are relatively divided. On the one hand, some people argue that this closure is important because it makes it possible to avoid gatherings: "there *were people who went to pray and as a result they caught the virus*" (H, 32, Dakar). But on the other hand, some people considered this closure to be irrelevant because they perceive that "*the pandemic is a divine will*, *it is a divine curse and naturally*, *in the face of these situations*, *the most favourable place to praise God are places of worship*, *which is why people could not accept their closure*" (H, 34, Tamba). The importance of the religious question will be seen later on.

### Perceptions of unexpected curfew effects

Qualitative evidence seems to show that the rather positive assessment of the curfew is less due to its ability to combat the pandemic, and more due to the production of unanticipated positive effects. S3 Fig shows that the perception of the curfew’s ability to reduce disease is not superior to other measures; indeed, it is perceived to be the least capable of doing so. Several people have even questioned this measure, questioning its relevance: "*I have the impression that people can only catch the disease if they travel from 8pm to 6am*" (F, 25, Dakar).

Thus, during the interviews, many explained that the curfew had in fact achieved other objectives such as education, strengthening security in neighbourhoods, family cohesion, and even peace in couples.

- *The importance of the curfew is not about illness but about educating people because there was indiscipline*" (H, 30, Kaolack).
- *There were less cases of theft*, *frankly*. There was *security*" (Female, 32, St-Louis)
- "*he had this setting where we ate together*. *Even the old people used to tell stories*" (H, 64, Dakar).
- *Some people have taken advantage of this to get closer to their families*" (Man, 34, Tambacounda).
- *a fluidity in the couple*" (H, 35 years old, Dakar)

### Coherence challenges regarding the removal of measures

**Fig 3:**
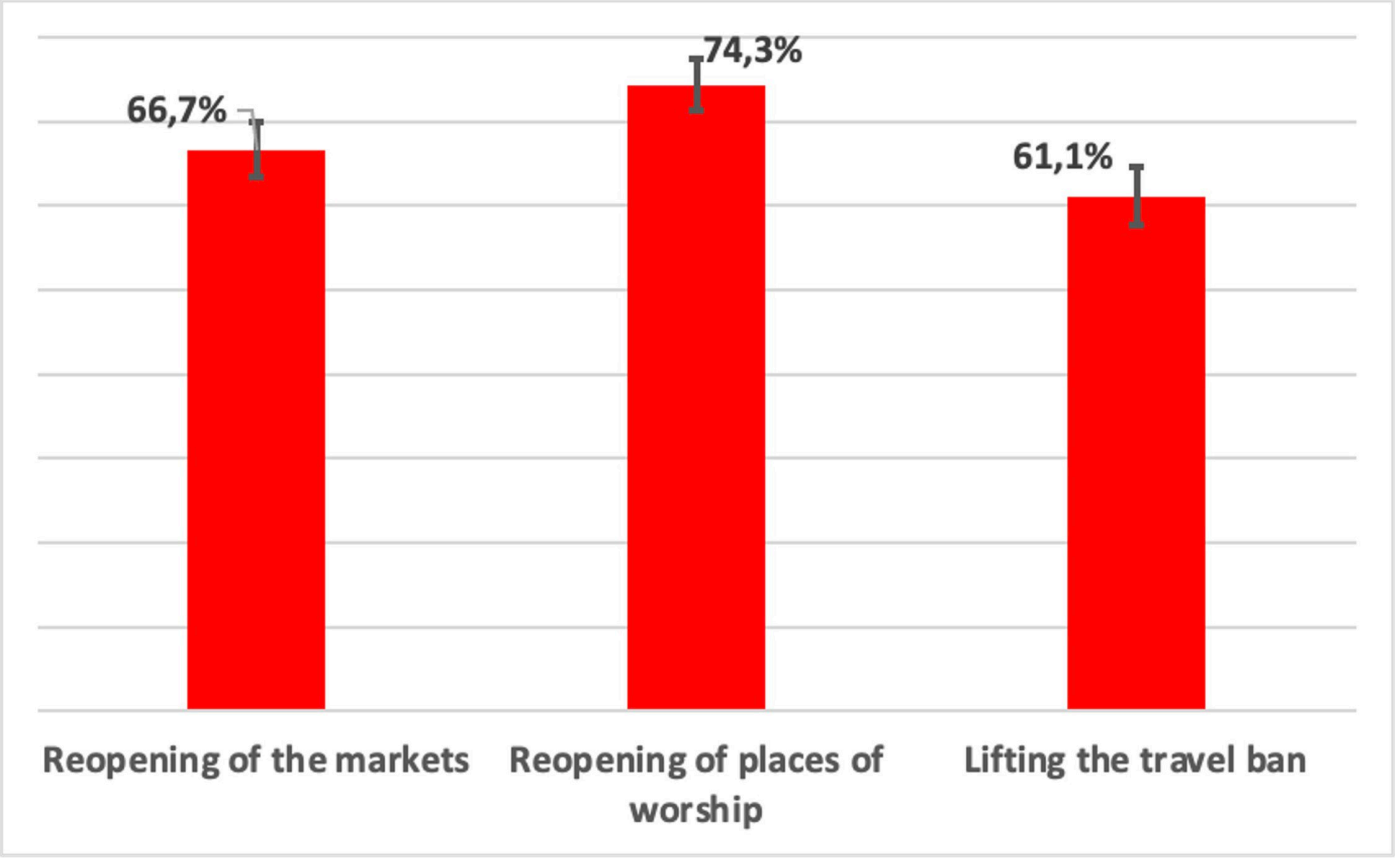
Agreement on the suspension of three measures.

As we have seen, Senegal has been very reactive in introducing drastic measures quickly to counter the spread of the pandemic [5, 25]. At the same time, however, some measures were only applied for a few weeks, less than two months. The study shows that, overall, the proportion of agreement with the removal of these measures is variable (Fig 3).

Qualitative data confirm this variable perception and show that many people had difficulty understanding the consistency of lifting these measures even though government communication affirmed their importance in curbing the pandemic. This was all the more difficult to understand given that at the time of the survey, new cases continued to be reported every day (Fig 1).

- *It was not important to close the places of worship if they should be reopened immediately because the threat for which they were closed is still there*. (H, 34, Tamba)
- *Yes*, *we noticed that the more the number of sick people increased*, *the more flexible the measures were*" (F, 42 years old, Thiès).
- *Yes*, *and it’s surprising because the minister addressed the people in a harsh tone and the next day or the same evening the president came out to say he was going to lighten up*. " (H, 40 years old, Thiès)

### Opposite generations

The quantitative results show significant contrasting perceptions between those under 25 and those over 60, except for the ban on movement between regions (Table 3). Apart from the ability to respect the closure of places of worship, which is comparable between the two age groups, for the other six dimensions of the acceptability of this measure, younger people are much less inclined than those over 60. The p-values in the last column are not the comparison of proportions, but the results of the test of the link between the age variable and the level of agreement with respect to each dimension of the measure.

**Table 3:** Acceptability of the four measures according to the two age groups.

	Under 25 years old			More than 60 years old			PVALUE
***Curfew from 9*:*00 pm to 5*:*00 am***	*Prop*.	*CI1*	*CI2*	*Prop*.	*CI1*	*CI2*	
*Importance*	83,8	78,4	88,0	95,7	87,5	98,6	**0,038**
*Efforts*	97,8	94,8	99,1	98,6	90,5	99,8	0,838
*Affective attitude*	83,3	77,9	87,6	95,7	87,5	98,6	**0,032**
*Disease reduction*	61,8	55,4	67,9	80,0	69,0	87,8	**0,019**
*Profits earned*	80,3	74,6	84,9	91,4	82,2	96,1	**0,093**
*Ability to respect*	95,2	91,5	97,3	97,1	89,3	99,3	**0,019**
*Consistency with personal values*	81,1	75,5	85,7	94,3	85,7	97,8	**0,009**
***Ban on travel between regions***							
*Importance*	77,6	71,8	82,6	82,9	72,2	90,0	0,157
*Efforts*	96,1	92,6	97,9	100,0	.	.	0,129
*Affective attitude*	73,2	67,1	78,6	84,3	73,8	91,1	0,169
*Disease reduction*	76,3	70,4	81,4	88,6	78,8	94,2	**0,023**
*Profits earned*	85,5	80,3	89,5	94,3	85,7	97,8	0,144
*Ability to respect*	95,2	91,5	97,3	100,0	.	.	0,162
*Consistency with personal values*	63,6	57,1	69,6	61,4	49,6	72,1	0,754
***Closing of the markets***							
*Importance*	68,4	62,1	74,1	84,3	73,7	91,1	**0,033**
*Efforts*	94,7	90,9	97,0	100,0	.	.	**0,038**
*Affective attitude*	63,2	56,7	69,2	87,1	77,0	93,2	**0,001**
*Disease reduction*	71,9	65,7	77,4	88,6	78,7	94,2	**0,013**
*Profits earned*	75,0	68,9	80,2	91,4	82,1	96,1	**0,004**
*Ability to respect*	94,3	90,4	96,7	98,6	90,4	99,8	0,094
*Consistency with personal values*	54,8	48,3	61,2	57,1	45,3	68,2	0,094
***Past closure of places of worship***							
*Importance*	52,6	46,1	59,0	71,4	59,8	80,8	**0,017**
*Efforts*	85,1	79,8	89,2	92,9	83,9	97,0	**0,063**
*Affective attitude*	50,0	43,5	56,5	72,9	61,3	82,0	**0,003**
*Disease reduction*	61,0	54,5	67,1	81,4	70,6	88,9	**0,005**
*Profits earned*	68,4	62,1	74,1	82,9	72,2	90,0	**0,060**
*Ability to respect*	90,4	85,8	93,6	92,9	83,9	97,0	0,553
*Consistency with personal values*	39,9	33,7	46,4	55,7	44,0	66,9	**0,064**

Similarly, older persons were the least in agreement with the removal of measures, whether it be the reopening of markets, places of worship, or movement between regions (S4 Fig). Moreover, older persons have full confidence in government to act against the pandemic, whereas younger persons are much more sceptical (S2 Table).

Qualitative data provide explanations for these differences in perception, which are sometimes diametrically opposed: "There is *a difference between the two visions*. There is a difference between the two visions" (H, 24, Dakar). Thus, the people we met confirm that the elderly are fully aware of their vulnerability to the pandemic, so much so that the message widely carried by the media pointed to the fact that the first victims were people of their own age. "*Older people are more aware*" (F, 70, Dakar). Moreover, although they are obviously still part of society, they are on the fringes of commercial or office activities, so measures to reduce these activities or travel hardly affect them. Thus, the cancellation of these measures worries them because they know that they are at risk. Moreover, in Senegal, older persons are often perceived as following the state and the government: "*Even in the political arena*, *older persons still trust the government in place*" (H, 36, Dakar). In contrast, younger people are connected to social networks and more inclined to discuss state decisions. Some young people are said to be in denial of the existence of the pandemic and view it as a conspiracy theory: "*Yes*, *it is because older people have more confidence in the state than we young people and adults do*. *We say that the disease is politicised*, *that they let us go to work*" (H, 25, Kaolack). One elderly person even stated, "*Already they say they don’t believe in the virus*. *Someone who doesn’t believe in the virus*, *how can he believe in the government*? *"*(F, 63, Thiès). Younger people are obviously the first to be affected by travel bans and their ability to travel for work.

### Confidence and pandemic: A regional and gendered opposition

**Fig 4:**
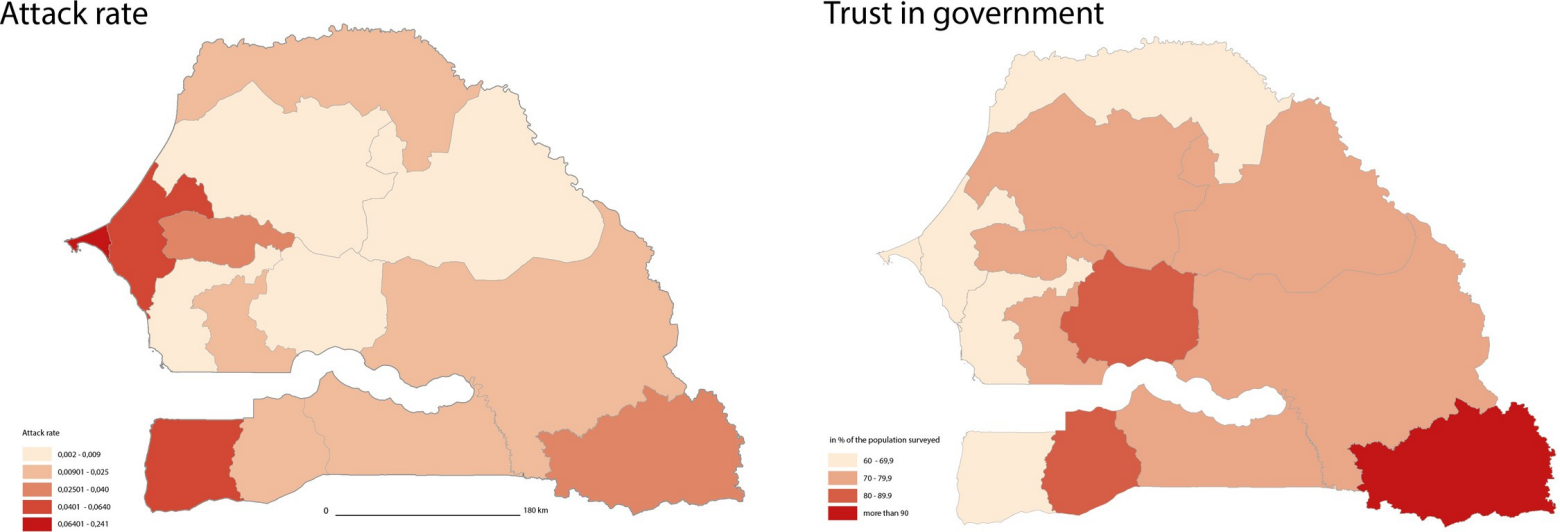
Regional distribution of attack rate and trust in government.

**Fig 5:**
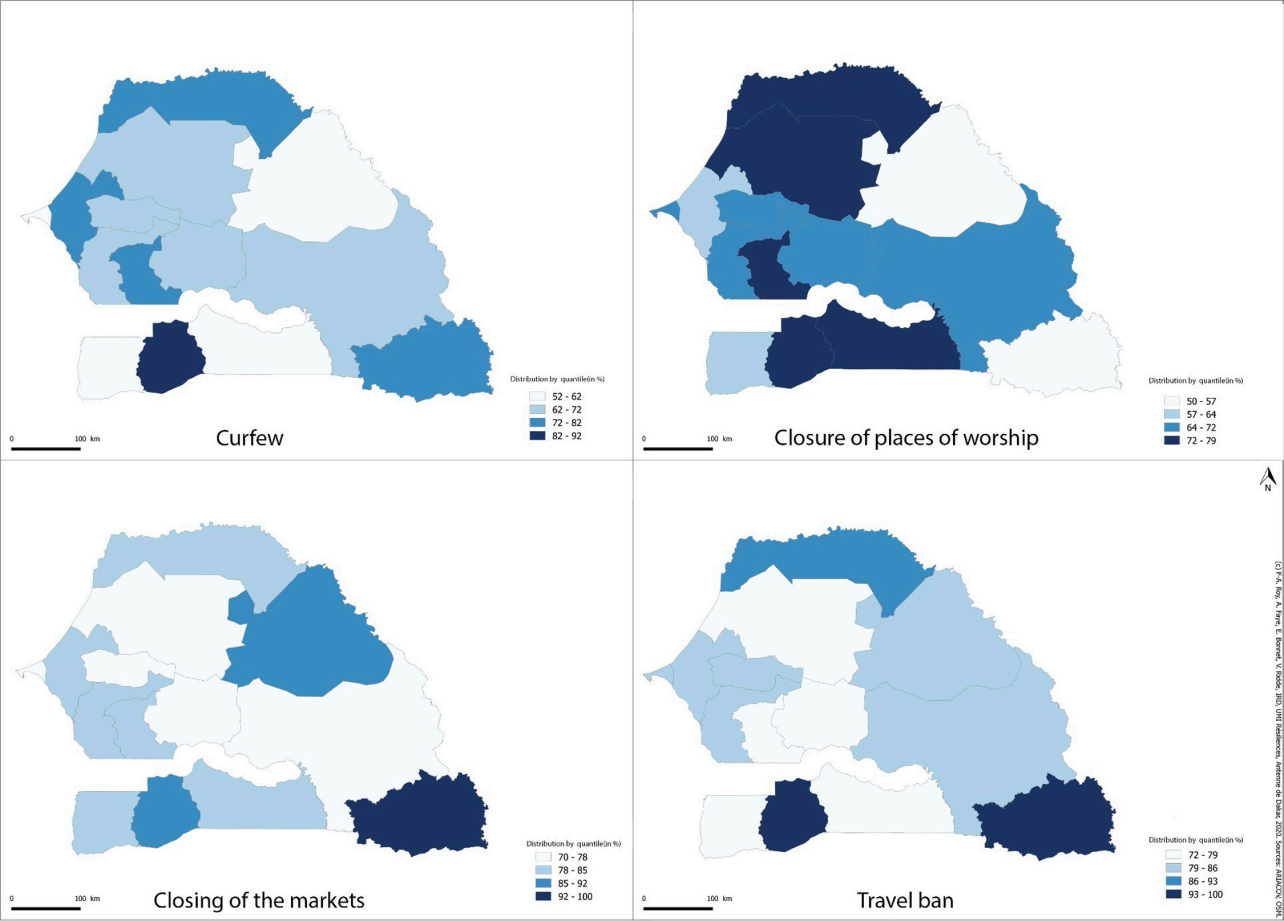
Regional distribution of perceived effectiveness of the four measures.

Fig 4 shows that the regions of Dakar, Thies, and Ziguinchor have the highest attack rates in the country (S5 Fig). On the other hand, the regions of Kédougou, Kaffrine, and Sédhiou bring together the respondents who have the most confidence in the government. This geographical distribution shows an inversely proportional relationship in the quantitative results between respondents’ confidence in the government to fight the pandemic, the perceived effectiveness of measures (except for the closure of places of worship, Fig 5) and the distribution of the attack rate between regions (Fig 4). While the average confidence level was relatively high (7.12/10), the higher the attack rate at the time of the survey, the less confidence people had in the government and the less effective they thought the measures were in combating the disease (one of the seven conceptual dimensions of acceptability). Spearman’s correlation between the average trust in government for each region and the rate per region is -0.24 (p = 0.39).

The qualitative results provide a better understanding of this relationship. The people we met believe that this difference can be explained in particular by the fact that despite the government’s actions, the pandemic was continuing to spread in the country. It is, therefore, partly the effectiveness of these measures that is at issue in the perceptions of a reverse trend between confidence and the development of the pandemic: "*For me it is because the state was fighting the disease and each time the number of contact cases increased*. *And so they told themselves that the measures taken were not effective*" (H, 20, Diourbel). Moreover, many people do not blame the state for the challenges of the effectiveness of its measures because they suggest a divine origin of the pandemic: "*But who can fight this disease*, *given that we don’t know where it comes from"* (H, 63, Thiès), "*This disease is the work of God*, *he will decide its end when the time is right*" (H, 36, Kaffrine).

Several people questioned the way the government communicated about the disease and its actions, undermining their trust in the state. Deficits in the communication process were often highlighted by respondents: "*The trust or lack of trust is explained by the information given by the government*. *Sometimes there are errors in the processing of information*, *which is why people will not trust 100%*. *"*(H, 51, Diourbel).

Beyond regional differences, quantitative data show that men trust the government less than women (6.84 ± 3.21 vs. 7.47 ± 3.05; *p value* = 0.001). Qualitative explanations confirm this opposition according to gender. The men tell us that it can be explained by the fact that the women are more passive: "*What explains this difference is that the man is more instigating; he will research the thing first before believing it*, *whereas the woman*, *when she is told something*, *she will be satisfied with it*. *But the man will always question it*. "(H, 51, Diourbel). They would even be more "*in acquiescence*" (H, 34, Tamba), without trying to understand the pandemic. Unsurprisingly, they argue that *"men are braver than women; for a small price*, *they frighten each other*. "(H, 18, Kaffrine). Moreover, men are said to have defied the state and its measures more than women "*almost all the people who demonstrated during the curfew are men*, *women rarely participate*. "(H, 25, Kaolack). Women are said to be more respectful of the guidelines than men. On the other hand, women believe that their greater confidence in the government is explained by the fact that they are "*smarter*" (F, 23, Dakar), more pragmatic, close to the reality of daily life and, above all, of the state agents and services with which they are more often in contact. Thus, "*men tend to question the decisions of the state; they say that the state does not tell the truth about the disease*. *On* the *other hand*, *it is the women who stay in the houses and follow what happens*" (F, 32, St-Louis). The "*politicisation*" of the pandemic by the state is often cited by women to justify the perception of men.

## Discussion

Based on a national telephone survey and a mixed-method approach using an appropriate conceptual framework [19], this study showed how the social acceptability of government measures to combat the COVID-19 pandemic in Senegal was highly variable. While the level of acceptability was generally satisfactory at the time of this study (June/July 2020), quantitative analyses highlighted differences according to age, gender, region, and also according to the measures organised by the state. The qualitative survey that followed was fruitful in understanding these differences and grasping the social context of their explanation, which quantitative measures cannot always highlight. Researchers have shown how the spread and explanation of the pandemic was heterogeneous and depended very much on the contexts [35]. It is also the organisation of universal measures in relation to interventions adapted to regional contexts, gender, or age that is referred to in these results. Many studies highlight the importance of adapting interventions to the contexts and people concerned in order for them to be effective [36]. However, this adaptation is obviously a challenge for governments in their desire to act for all their citizens.

In Senegal, another telephone survey carried out in early April 2020 showed that 86% of people were very or rather confident in the government’s ability to take care of its citizens without any gender or regional differences [37]. Another telephone survey conducted only in the capital (Dakar) in early April showed that 88% of people were very or somewhat satisfied with government measures. Similarly, 86% of Dakar residents were in favour of the closure of places of worship without any known differences according to age and gender [38]. These differences with our survey, where confidence levels are relatively lower, could be explained by the fact that it took place more than two months later, thus giving respondents time to notice the spread of the virus (Fig 1) despite government measures. Another national telephone survey in Senegal conducted in August 2020, just after our survey, confirms our findings by showing that men are less satisfied with the government response than women (52% vs. 60%). Contrary to our study, they did not find any difference between the youngest and the oldest regarding this level of satisfaction (62% vs. 64%) [39].

Elsewhere in the world, research shows the importance of the role of trust in the state for the acceptability of measures taken to fight the pandemic [24, 40]. Several studies (and a review [41]) amid the pandemic in Europe, the USA, and China have confirmed the importance of citizens’ trust in governments to increase their acceptance and adherence to non-pharmaceutical interventions [8, 9, 24, 42]. Conceptually, trust is one of the important determinants of acceptability [43]. In Nigeria, mistrust of the state and its forms of corruption play a negative role in the acceptance of measures to combat the COVID-19 pandemic [44] especially since in the same West African country and in Egypt, two other studies show that respondents believe that the states are not doing enough to curb the pandemic [16, 17]. The fight against Ebola in West Africa has also shown how central the issue of trust is in the acceptability of control strategies [45]. In Senegal, several of our respondents denounced a politicisation of the management of the pandemic, as seems to be the case in West Africa [5] and elsewhere [46, 47]. The management of the HIV epidemic in Africa had also given rise to such analyses [48].

Beyond the negative effects of curfews on health noted in Senegal [49] and Kenya [50] example, our study showed that while the perception was very positive for the majority of people, it was not necessarily to fight the pandemic. The qualitative aspect showed that the curfew was appreciated for its unintended effects: security and social/family cohesion. The analysis of the unintended effects of public health interventions is rarely carried out, particularly in Africa [51]. In this case, it is thanks to our qualitative approach that they have been brought to light. This result shows the value of mixed methods and qualitative studies for understanding complex situations. The analysis of the unexpected effects of the measures taken by States to combat the pandemic remains to be done [52]. While in Senegal the unintended effects perceived by respondents are essentially positive, work is beginning to show some negative unintended effects of government responses, particularly for the most vulnerable people or in low-income countries [53–55]. Here we are obviously thinking of economic issues, but also mental health issues.

On the other hand, a relatively expected perception was that of gender difference. The results of the study confirm gender stereotypes and their integration into thought patterns. Men are rebellious and strong, women are sensitive and confident (and in their opinion intelligent). It is essential to take gender into account in the fight against the pandemic [56] particularly in West Africa [57]. While the quantitative survey was carried out by women, the qualitative survey was carried out by a man, which may not have made it possible, particularly over the telephone, to deepen this gendered vision of perceptions. Further analysis of our data in this regard is planned in a future article.

A survey carried out in Senegal in August 2020 showed that 49% of the over-56s say they have not been to their place of worship in the last seven days compared to 33% of the under-25s [39], confirming the perception of our study according to age. The question of closing places of worship and then reopening them seven weeks later was certainly the most discussed by the respondents in our research. Religion is a very important social issue in Senegal. As has been shown in Europe and elsewhere, religion is a factor that should not be neglected in the fight against the COVID-19 pandemic [58]. This issue affects people’s values but also the way in which the Senegalese state governs its relations with Muslim brotherhoods and Catholic authorities. Older people were most in agreement with their closure, and then disagreed with their reopening. Since they are the people who frequent places of worship the most, one might have thought the opposite. This was not the case because older persons are genuinely aware that they are most at risk in the context of the COVID-19 pandemic, especially when one considers their challenges in terms of access to care [59], intergenerational solidarity [60] in Senegal, and the difficulties in applying measures in places of worship. The average age of serious cases has been estimated at 64 years in Senegal, with lethality ranging from 41% to 45% [25]. This perception of their vulnerability acquired in a few months is remarkable and can be a positive element in terms of communication about the pandemic. In France, the youngest populations are most resistant to the State measures [61]. As in the case of the analyses concerning the spread of HIV on the continent [48], it could be hypothesised that, although susceptibility to the COVID-19 pandemic appears to be lower than elsewhere in the world [62] for reasons which remain to be explained, the vulnerability of the elderly is obvious and perceived as such by those concerned. Future research should ask, following the theoretical propositions of the HIV analysis, if this low population susceptibility in Senegal could be explained by strong social cohesion in a low-income country [48]?

On the other hand, like others, they did not understand the lifting of these measures when the pandemic was still going on and was constantly being discussed in the media. The challenges of policy coherence were not limited to places of worship, as a Senegalese social scientist notes [63]. They also emerged when it was announced that schools would reopen for examination classes on June 2, cancelled on June 1st and finally authorised on June 25. Then the Senegalese President, who was himself in quarantine, announced the lifting of the state of emergency and curfew on June 29 only 24 hours before, leaving the population sceptical about this swift decision. In addition, as in Côte d’Ivoire, displacement measures for teachers returning to Senegalese regions were lifted on June 4 (in the midst of a pandemic, see Fig 1), with the immediate consequence of the virus circulating. We are, therefore, at the heart of the study of the coherence of public policies, a new criterion for evaluating development interventions [64], and a central concept in political science [13, 65]. The national press has also widely commented on the state’s decision to authorise the reopening of places of worship, claiming that the justification was in no way health-related, but political, as it gave way in particular to pressure from Muslim brotherhoods, which are known to have enormous political and economic weight in Senegal [63]. The Catholic community decided not to follow this directive, as did some brotherhoods who "*refused to close their mosques and continue collective prayers*" [63]. Denial of the pandemic was already noted by anthropological research at the beginning of the pandemic in these regions [66]. The great annual pilgrimage of Touba, bringing together more than four million people in October 2020 in these same regions, has not been cancelled [67] even though this region was one of the first clusters of the pandemic [5]. Obviously, the economic stakes of these decisions cannot be denied in countries where the informal and agricultural sectors are very important. A national survey by ANSD in July 2020 in Senegal showed that 85% of households claimed to have suffered a drop in income, and 36% of heads of household said they had stopped working: 30% of them for reasons linked to the pandemic [68].

The strength of this study is that it used a nationally representative sample of quota criteria whose quantitative results were explained by a qualitative survey, using a sound conceptual approach [19]. The technical innovation of a telephone survey in a country without a national telephone directory is particularly noteworthy and will be the subject of another article. The study showed its feasibility, particularly in a pandemic context where it was a question of respecting barrier measures. The first limitations concern the fact that in phone surveys it is not easy to obtain many socio-economic variables. Thus, we do not have, for example, variables on the occupation or employment, which may be a limitation in interpreting the data to compare the acceptability of government measures to older and younger people. The secund limitations concern the statistical analyses, which remain relatively descriptive for the moment, but will be explored in greater depth for a future article. Multivariate analyses like structural equations would have provided a better understanding of the complex relationships between the variables studied. However, this relative simplicity was an essential condition for carrying out a rapid in-depth qualitative survey, so as not to increase the memory bias of the participants. If we had waited for the multivariate analyses, we would have lost the opportunity to have data to understand these results and inform decision makers. This trade-off is a classic challenge when using mixed methods. We will publish further multivariate analyses in a forthcoming paper, as well as a psychometric analysis of the TFA instrument, the exploratory results of which are encouraging. Furthermore, the third limitations, due to a lack of resources and the fact that the governmental measures were not implemented for a long time, we were not able to carry out a longitudinal study to study the evolution of their acceptability by the population.

## Conclusion

This research in Senegal confirms the importance of the quality of government communication and the trust that a state should engender in order to strengthen the acceptability of the measures taken against the pandemic and thus their potential effectiveness. Moreover, significant differences in acceptability between regions, ages, and gender highlight the essential need to adapt measures and, above all, their explanations, instead of universal action that does not take into account contexts and individuals.

## Supporting information

S1 Table Responses before and after the rewording of the question on the acceptability of curfews. (DOCX)

S2 Table Trust in government by age (0 to 10). (DOCX)

S1 Fig Concordance with personal values concerning the 4 measures. (DOCX)

S2 Fig Positive affective attitude about the 4 measures. (DOCX)

S3 Fig Perceived effectiveness of the four measures in reducing the disease. (DOCX)

S4 Fig Agreement to suspend measures according to the age. (DOCX)

S5 Fig Map of the regions of Senegal. (DOCX)

S6 Fig Flowchart. (DOCX)

S1 Text Interview guideline questions. (DOCX)
