# Supplementary material for: Acceptability of government measures against COVID-19 pandemic in Senegal: A mixed methods study
Source: PLOS Glob Public Health. 2022 Apr 25;2(4):e0000041. doi: 10.1371/journal.pgph.0000041 (PMC10021345; doi:10.1371/journal.pgph.0000041)
Supplement: S1 Table — (DOCX) [file pgph.0000041.s001.docx]

S1 Table: Responses before and after the rewording of the question on the acceptability of curfews

|  | **Before (N=586)**  **N, (%),** [**CI**]. | **After (N=227)**  **N, (%),** [**CI**]. | **P Value** |
| --- | --- | --- | --- |
| Importance | 490 (83,6%)  [80,4%-86,4%] | 207 (91,1%)  [86,7%-94,5%] | **0,008** |
| Efforts | 570 (97,2%)  [95,6%-98,3%] | 224 (98,6%)  [96,2%-99,7%] | 0,350 |
| Affective attitude | 497 (84,8%)  [81,7%-87,5%] | 203 (89,4%)  [84,7%-93,1%] | 0,111 |
| Perceived effectiveness | 371 (63,3%)  [59,3%-67,1%] | 166 (73,1%)  [66,9%-78,8%] | **0,010** |
| Benefits drawn | 478 (81,6%)  [78,2%-84,5%] | 197 (86,7%)  [81,7%-90,9%] | 0,094 |
| Ability to respect | 575 (98,1%)  [96,7%-98,9%] | 218 (96,0%)  [92,6%-98,2%] | 0,141 |
| Consistency with personal values | 504 (86,0%)  [83,0%-88,6%] | 197 (86,8%)  [81,7%-90,9%] | 0,861 |
