# Supplementary material for: Acceptability of government measures against COVID-19 pandemic in Senegal: A mixed methods study
Source: PLOS Glob Public Health. 2022 Apr 25;2(4):e0000041. doi: 10.1371/journal.pgph.0000041 (PMC10021345; doi:10.1371/journal.pgph.0000041)
Supplement: S2 Table — (DOCX) [file pgph.0000041.s002.docx]

S2 Table: Trust in government by age (0 to 10)

|  | **Staff** | **Average** | **Difference Type** | **Median** | ***P value*** |
| --- | --- | --- | --- | --- | --- |
| **Under 25 years old** | 228 | 7,07 | 3,11 | 8,0 | 0,10 |
| **25-59 years old** | 515 | 7,06 | 3,17 | 8,0 |  |
| **60 years old and over** | 70 | 7,72 | 3,12 | 9,0 |  |
