# Supplementary figures and images for: Acceptability of government measures against COVID-19 pandemic in Senegal: A mixed methods study
Source: PLOS Glob Public Health. 2022 Apr 25;2(4):e0000041. doi: 10.1371/journal.pgph.0000041 (PMC10021345; doi:10.1371/journal.pgph.0000041)

S1 Fig: Concordance with personal values concerning the 4 measures


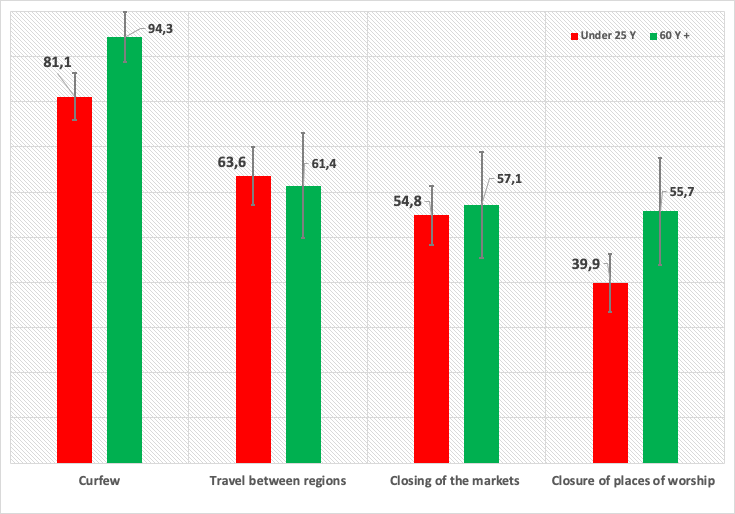

Supplement: S1 Fig — (DOCX) [file pgph.0000041.s003.docx]

S2 Fig: Positive Affective attitude about the 4 measures


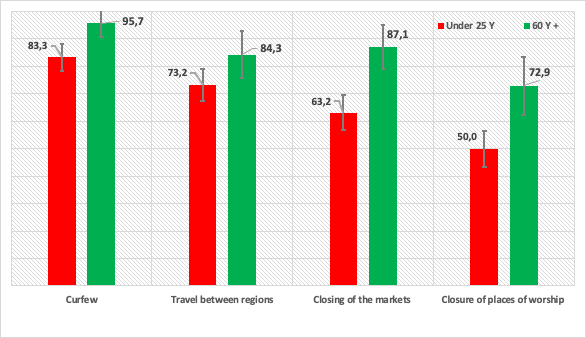

Supplement: S2 Fig — (DOCX) [file pgph.0000041.s004.docx]

S3 Fig: Perceived effectiveness of the four measures in reducing the disease


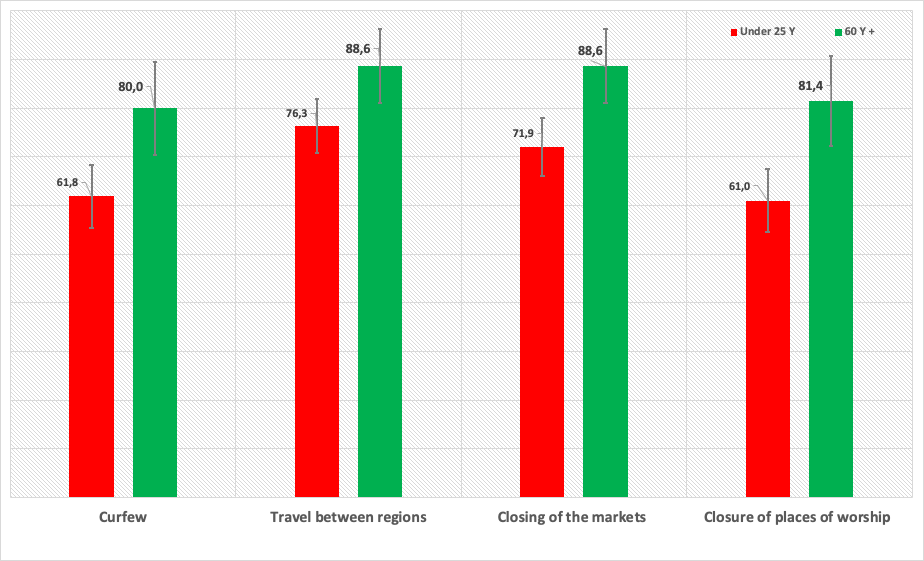

Supplement: S3 Fig — (DOCX) [file pgph.0000041.s005.docx]

S4 Fig: Agreement to suspend measures according to the age


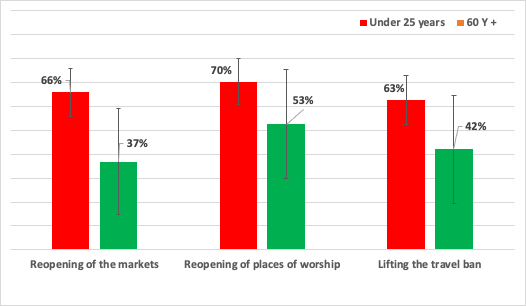

Supplement: S4 Fig — (DOCX) [file pgph.0000041.s006.docx]

S5 Fig: Map of the regions of Senegal


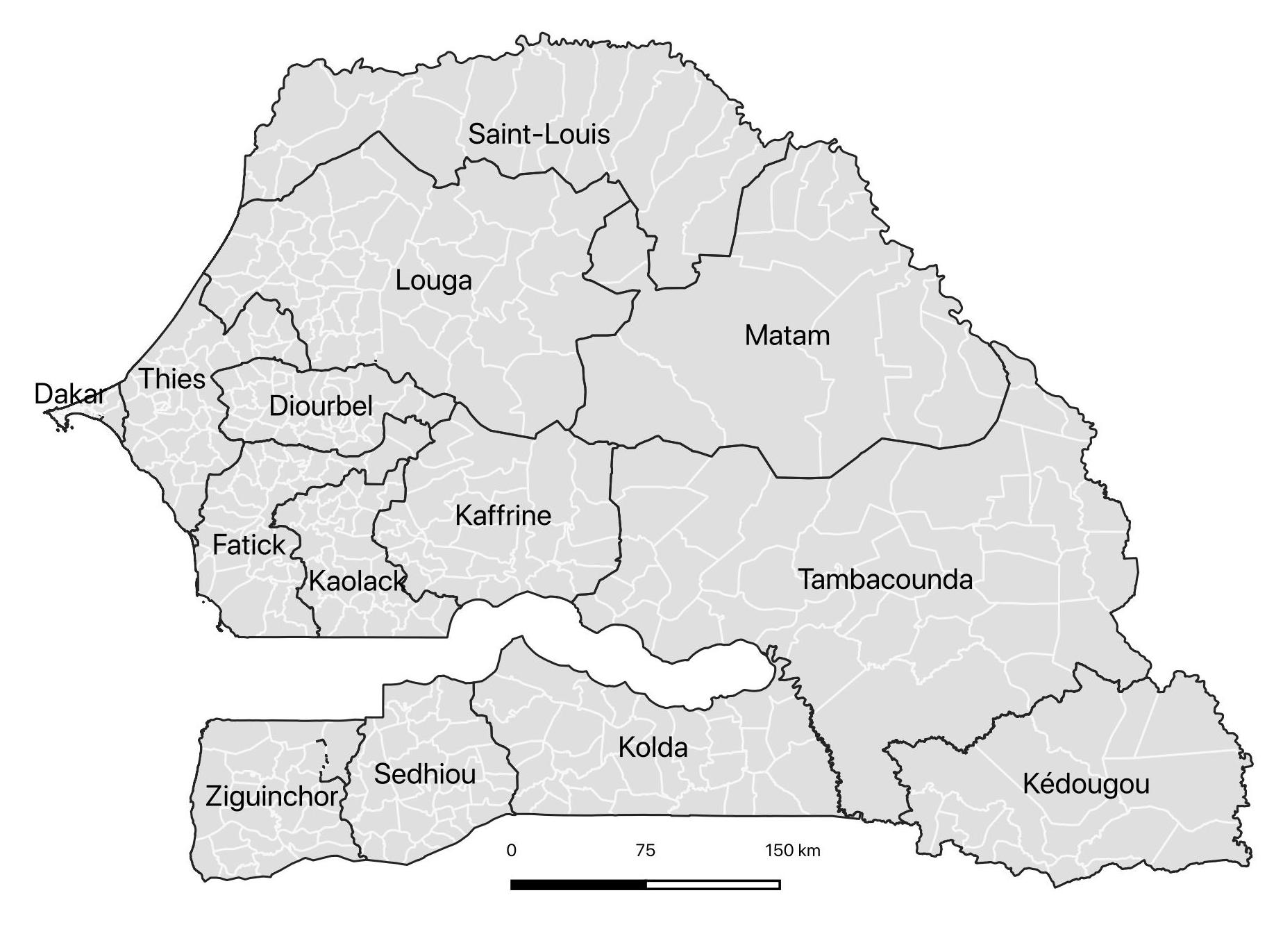

Supplement: S5 Fig — (DOCX) [file pgph.0000041.s007.docx]
