# Supplementary material for: Acceptability of government measures against COVID-19 pandemic in Senegal: A mixed methods study
Source: PLOS Glob Public Health. 2022 Apr 25;2(4):e0000041. doi: 10.1371/journal.pgph.0000041 (PMC10021345; doi:10.1371/journal.pgph.0000041)
Supplement: S6 Fig — (DOCX) [file pgph.0000041.s008.docx]

S6 Fig: Flowchart

Detected as human or not sure by the platform

N = 1,441

Excluded

N = 628

Included

N = 813

Answering machine or quick hangup

N = 5,135

Outgoing call no picked up

N = 4,355

Outgoing call picked up

N = 6,576

List of unique telephone numbers randomly selected (Random Digit Dialing)

N = 30,603

List of numbers assumed to be valid

N = 10,931

Invalid Numbers

N = 19,672
