# Supplementary material for: Acceptability of government measures against COVID-19 pandemic in Senegal: A mixed methods study
Source: PLOS Glob Public Health. 2022 Apr 25;2(4):e0000041. doi: 10.1371/journal.pgph.0000041 (PMC10021345; doi:10.1371/journal.pgph.0000041)
Supplement: S1 Text — (DOCX) [file pgph.0000041.s009.docx]

S1 Text: Interview Questions

In the fight against the pandemic, people have understood the importance of curfews much more than the importance of closing places of worship. What do you think about this?

The older people are, the more they think that the measures taken will reduce the disease. What do you think about this?

In areas where the disease is more prevalent, people have less confidence in the government to fight the epidemic. What do you think about this?

The majority of people trust the national media and health professionals to provide information about the coronavirus much more than social media. What do you think about this? How do you explain this when everyone is following these social media?

Men are less confident in the government's ability to act on the pandemic than women. What do you think about this? How can you explain this difference?

Regardless of age, men tend to be less worried about the epidemic than women. What do you think about this? How can you explain this difference?

Older people

The older people are, the more they understand the importance of closing places of worship and their disagreement with their reopening. What do you think about this?

The older people are, the more they understand the importance of all the measures, and this is especially true for understanding the importance of closing places of worship and their disagreement with reopening them

The older people are, the less they agree with the reopening of travel between regions. What do you think about this?

Older people have full confidence in the government to act against the pandemic, unlike younger people or adults. What do you think about this? How do you explain this difference?
